# Comparison between olfactory bulb and olfactory tract implantations through an endoscopic supraorbital keyhole approach: a cadaveric study

**DOI:** 10.1007/s00701-025-06752-9

**Published:** 2025-12-22

**Authors:** Hakim Benkhatar, Douglas Henderson, Teofil Mures, Claire Martin, Damien Bresson

**Affiliations:** 1 Centre Hospitalier de Versailles, Service d’ORL Et Chirurgie Cervico-Faciale, 177 Rue de Versailles, 78150 Le Chesnay-Rocquencourt, France; 2 Hôpital Foch, Service de Neurochirurgie, Suresnes, France; 3 Ecole de Chirurgie du Fer À Moulin, Paris, France; 4 Université Paris Cité, BFA, UMR 8251, CNRS, 75013 Paris, France; 5 Université Paris-Saclay, UVSQ, 78180 Montigny-Le-Bretonneux, France

**Keywords:** Olfactory loss, Olfactory implant, Olfactory bulb, Olfactory tract, Neurostimulation, Supraorbital keyhole craniotomy

## Abstract

**Purpose:**

Since the COVID-19 pandemic, olfactory loss has been recognized as a highly prevalent condition that greatly impacts the quality of life. Similar to other sensory implants, the idea of an olfactory implant has emerged. Evidence indicates that electrical stimulation of specific olfactory structures can evoke smell sensations. However, debates on the most appropriate anatomical target and surgical technique for implantation are still ongoing. By extrapolating data from other surgical indications, transcranial approaches appear to carry a lower risk of cerebrospinal fluid leakage and infection compared with endoscopic endonasal routes. The aim of this study was to compare two electrode placements (dorsal olfactory bulb and ventral olfactory tract) through a supraorbital keyhole craniotomy in human cadavers.

**Method:**

Four fresh human cadavers were dissected in a staged manner. Supraorbital keyhole craniotomy was performed through an eyebrow incision and the frontal lobe was slightly retracted to allow angled (30°) endoscope insertion. An auditory brainstem implant (ABI) from MED-EL was used for electrode placement.

**Results:**

Endoscopic placement of the electrode on the dorsal side of the olfactory bulb was achieved after orbital roof drilling in all cases, but was not stable. On the contrary, endoscopic placement of an electrode under the olfactory tract was easily achieved without drilling and the electrode was stable between the olfactory tract and the planum sphenoidale, behind the olfactory bulb.

**Conclusion:**

Ventral olfactory tract implantation posterior to the olfactory bulb using an ABI is straightforward and is associated with satisfactory electrode stability. Such a procedure could be used for clinical pilot studies evaluating the effects of various stimulation protocols on the olfactory tract in patients with long-lasting olfactory loss.

## Introduction

Olfactory loss prevalence and awareness have greatly increased since the COVID-19 pandemic [17]. Olfactory loss greatly impacts quality of life in many aspects. It decreases daily pleasure related to food intake, it decreases personal safety as lacking the ability to detect a gas leak, a burning smell or spoiled food, increases the risk of hazardous events and impair social behavior [12, 20]. Depression is therefore highly prevalent in this population [23]. Therapeutic research in this field has also greatly increased after the pandemic, and two research focuses have emerged: electrical stimulation of the olfactory system and olfactory regenerative therapies [13]. Regarding the first research field, olfactory implants are currently being developed by multiple teams around the world [2, 14]. These electronic devices basically comprise a gas sensor (or electronic nose) capable of odor identification and an electrical stimulator including a surface electrode array in contact with a relevant section of the olfactory path: the olfactory mucosa (OM), the olfactory bulb (OB) or central olfactory networks such as the piriform cortex (PC) [15, 22, 34]. Evidence indicates that electrical stimulation of neural targets can evoke olfactory sensations; however, proof-of-concept studies assessing the outcomes of stimulating these targets remain limited, justifying the development of surgical techniques for olfactory implantation [34].

In case of loss or injury of olfactory receptor neurons (ORNs), as encountered in post-viral anosmia or post-traumatic anosmia related to severed olfactory filaments, the OB has been depicted as an attractive target for neurostimulation as, contrary to the PC, its superficial layer (glomerular layer) is functionally organized in different zones responding to specific odorants (a feature termed chemotopy) and its surgical access is less demanding, particularly through a supraorbital keyhole approach (SKA) [3, 7]. From an ethical standpoint, *non-lesional* (i.e. preserving the integrity of the OB) and *reversible* olfactory implantation is highly desirable for any future clinical pilot study on this topic: electrode placement on the *dorsal side of the OB* is therefore preferable in a first stage, as placing an electrode array under or even around the OB would require its detachment from the cribriform plate, annihilating any residual olfactory function on this side and even hindering other future treatment strategies such as olfactory regenerative therapies.

**Fig. 1:**
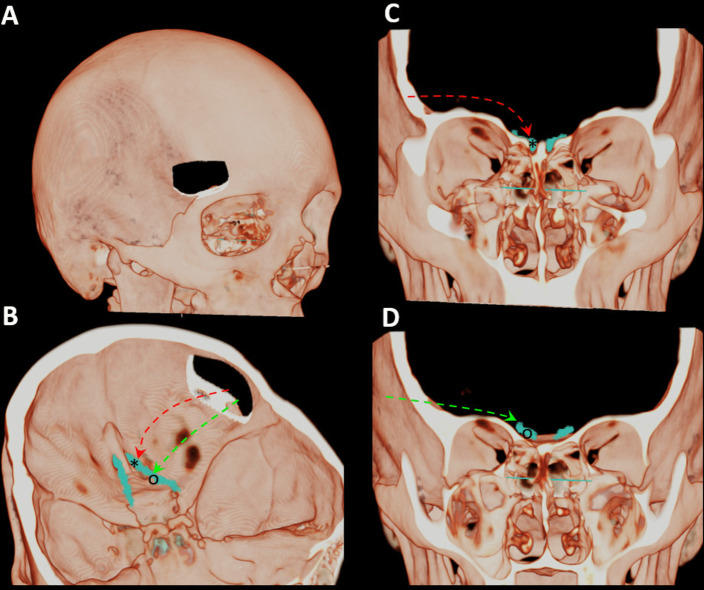
Illustration of supraorbital approach to the homolateral olfactory bulb and tract. **A** supraorbital craniotomy, **B**, **C**, **D** Access (red arrow) to the olfactory bulb (*) and access (green arrow) to the olfactory tract (^O^)

However, placing and stabilizing an electrode on the dorsal OB might be difficult for multiple reasons: its shape and size are variable [35], its depth in the olfactory fossa is variable [1], olfactory artery branches might constitute an obstacle [11] and the cerebrospinal fluid (CSF) flow in the olfactory cistern might lead to electrode displacement. Therefore, in this study, we intended to explore a new target, the olfactory tract (OT), which essentially contains the axons of secondary olfactory neurons whose cell bodies are located in the OB (mitral and tufted cells), which are spatially organized [19, 30]. We compared two electrode placements through SKA in human cadavers (Fig. 1): one on the dorsal OB and one on the ventral OT. Our hypothesis is that a ventral OT implant (OTI) might confer an easier, more stable implantation and less aggressive approach regarding the OT itself compared to an OB implant (OBI).

## Material and methods

**Fig. 2:**
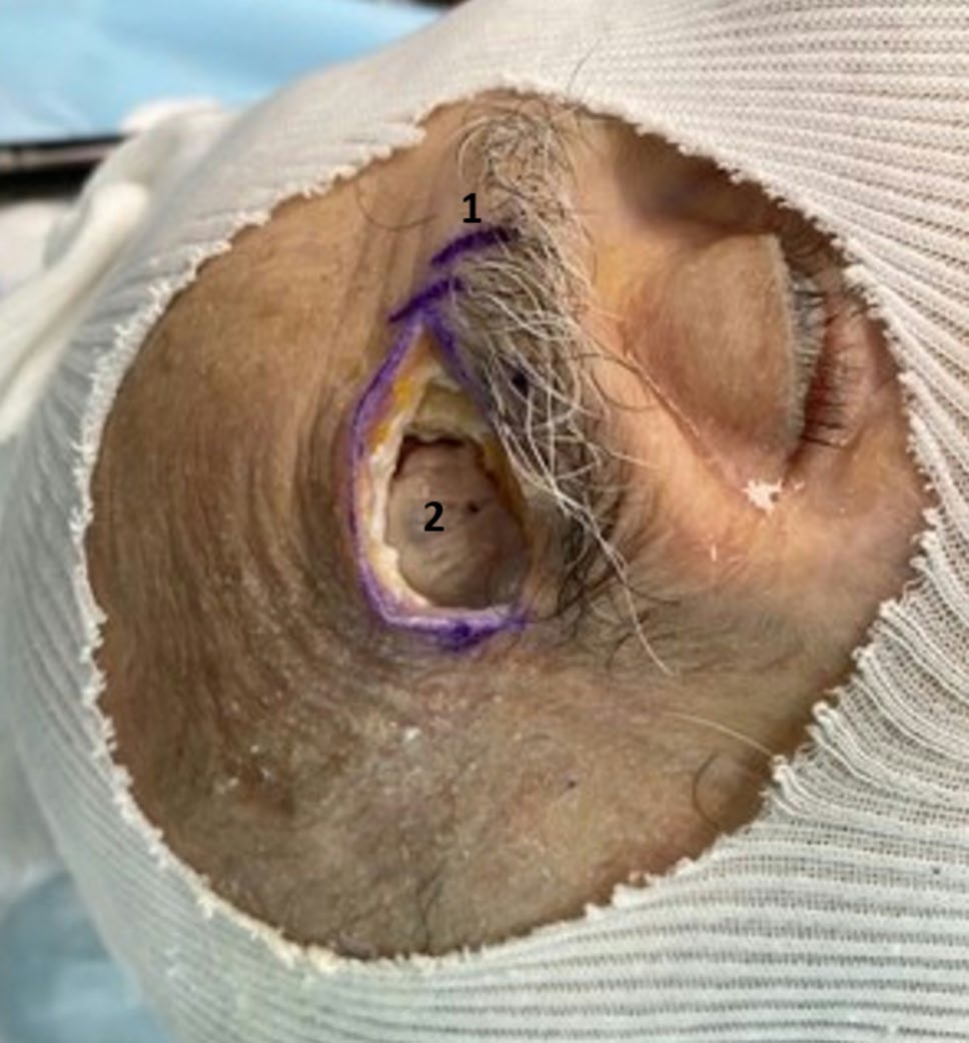
Supraorbital keyhole approach through a suprabrow incision. *1: Supraorbital notch outlined, 2: Craniotomy exposing the dura mater*

**Fig. 3:**
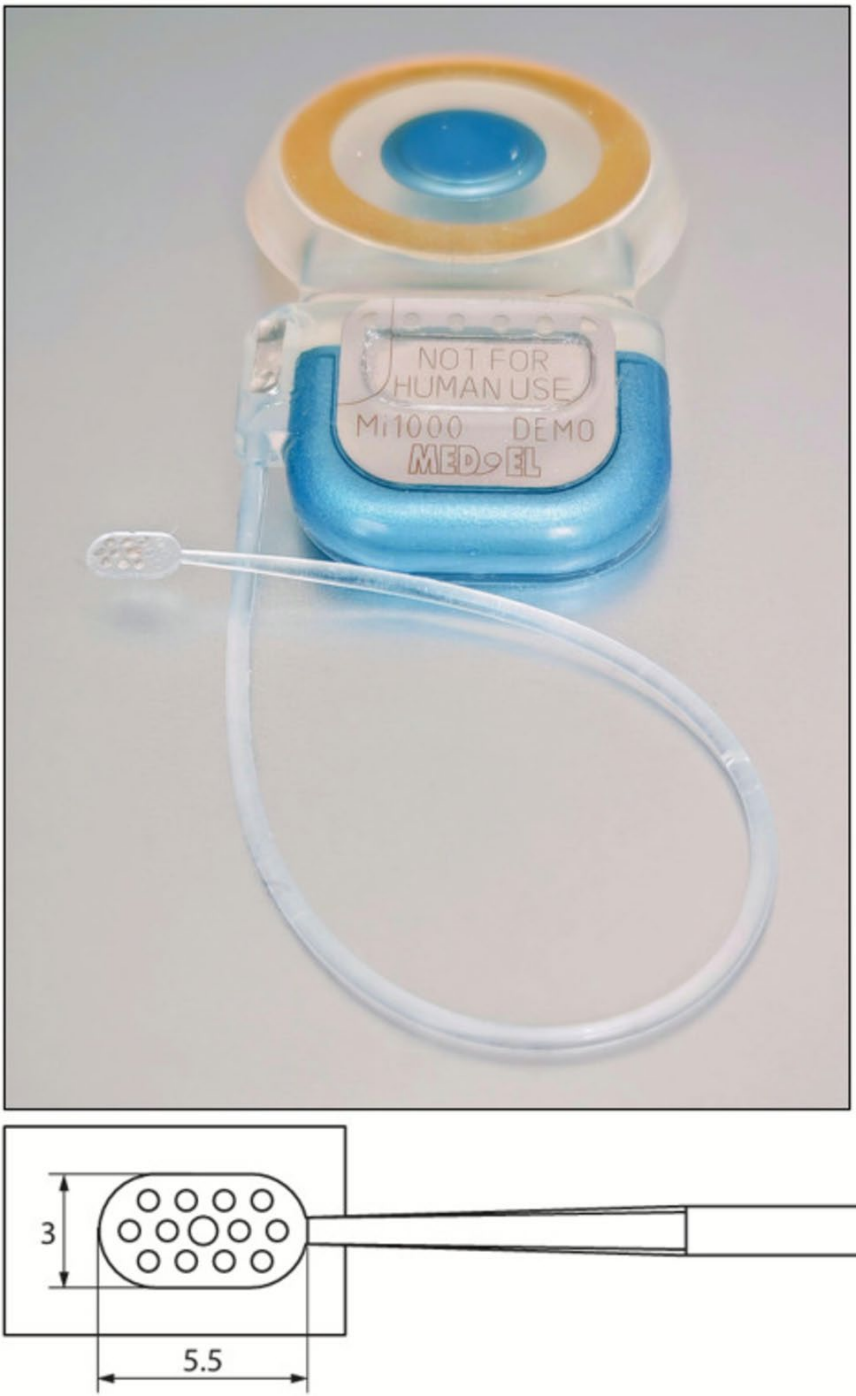
Mi1000 DEMO auditory brainstem implant (MED-EL) and its 12-contact electrode array (measuring 5.5 x 3 mm)

Four fresh human cadavers (ages ranging from 40 to 75 years old) were injected with dyes to allow visualization of arteries and veins and dissected in a staged manner. All surgeries were performed on the right side in 3 subjects and on both sides in 1 subject, by either DB or TM with the assistance of HB. After performing a 4 cm suprabrow incision lateral to the supra-orbital nerve, subperiosteal dissection and temporal muscle reclination were achieved behind the fronto-zygomatic suture to expose the area above and below the linea temporalis, up to the pterion. Then, a craniotome was used to achieve a 2 × 3 cm craniotomy above the level of the orbital roof, while preserving the integrity of the dura (Fig. 2). A U-shaped dural incision was made and the dural flap reclined and secured by stitches. Gentle frontal lobe elevation with a brain retractor allowed the insertion of an angled endoscope (30°) to search for the OT and the OB anteriorly. Finally, electrode placement on the dorsal OB and on the ventral OT (just behind the OB) was attempted using a Scoville alligator applying forceps (straight 2 mm) under endoscopic vision. A dummy auditory brainstem implant (Mi1000 DEMO) from MED-EL (Innsbruck, Austria) was used for implantation (Fig. 3), as the dimensions of the 12-contact grid electrode (5.5 × 3 mm) fitted the OB surface (11 × 4 mm on average) and the OT width (5 mm anteriorly behind the OB) [24]. This surgical protocol had approval from the ethical comity of the Fer à Moulin School of Surgery.

## Results

### Olfactory bulb implantation

**Fig. 4:**
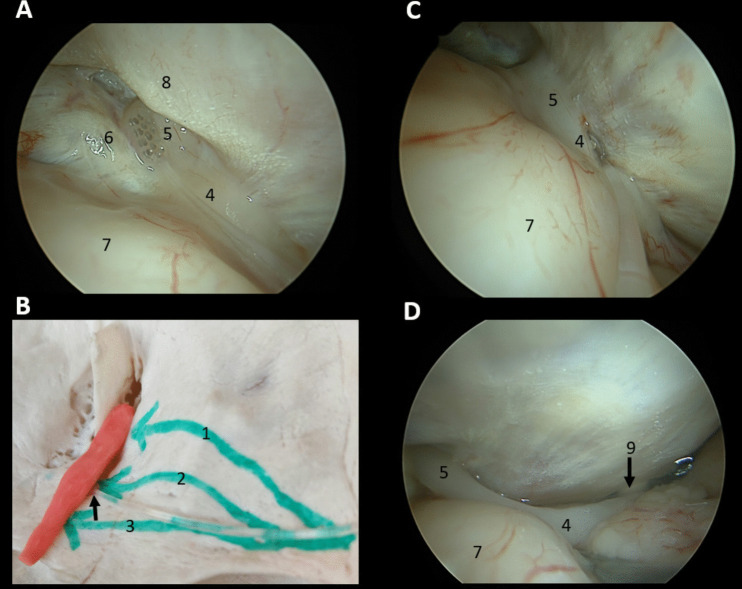
Olfactory bulb and tract implantation. **A** Endoscopic placement of the electrode on the dorsal olfactory bulb, **B** Illustration of corridors (green arrows) from the supraorbital craniotomy to the olfactory bulb and tract. An ABI electrode (black arrow) is placed under the anterior olfactory tract, **C** Endoscopic placement of the electrode under the anterior olfactory tract, **D** Olfactory tract in contact with the optic nerve posteriorly. *1: Corridor to the olfactory bulb, 2: Corridor to the anterior olfactory tract, 3: Corridor to the intermediate olfactory tract, 4: Olfactory tract, 5: Olfactory bulb, 6: Crista galli, 7: Frontal lobe, 8: Orbital roof, 9: Optic nerve*

Successful access and implantation to the dorsal OB was achieved in all cases (Fig. 4A). However, drilling of the orbital roof (along the corridor 1 in Fig. 4B) was necessary in all cases to allow proper access and visualization of the OB. No orbital fat extrusion occurred and no olfactory artery branches prevented access to the OB. However, electrode stability was poor, as no structure above the electrode could maintain it and any slight rotation of the electrode carrier between fingers would displace the electrode.

### Olfactory tract implantation

Successful access and implantation to the OT was achieved in all cases (Fig. 4C). Drilling of the orbital roof was never required, thus reducing operative time, as the OT was reached by following a straight line along the lesser wing of the sphenoid bone, as illustrated in Fig. 4B. The electrode was placed under the mid-portion of the OT and then gently pushed anteriorly with a smooth elevator, until covered by the anterior OT (Fig. 4C). Its stability between the anterior OT and planum sphenoidale was satisfactory in all cases, even if the electrode carrier was slightly rotated between fingers. As the posterior OT is in close contact with the optic nerve, electrode placement was not intended in that area (Fig. 4D).

## Discussion

Neurostimulation of the olfactory system has recently attracted growing interest from scientific communities working on olfactory loss [34], but also on conditions potentially associated with olfactory loss such as depression [21] and cognitive decline [31]. Indeed, the functional outcome of OB or OT stimulation have been evaluated in both pre-clinical and clinical studies. Many studies in rodents have shown that odorants activate the OB according to a specific spatio-temporal pattern [27] and Coelho et al. showed that electrical stimulation of the OB can reproduce spatial and temporal dimension of neural activity [4, 5]. Moreover, in humans, both subdural and transethmoidal electrical stimulation of the OB can induce the perception of smell [14, 18]. Patients eligible for such intervention should have at least 3 characteristics: (i) a significant olfactory loss (hyposmia or anosmia) severely impacting the quality of life, (ii) an MRI showing a preserved olfactory anatomy on one side at least (with no or minor injury to the OB, OT and olfactory cortices) and (iii) a lack of effectiveness for all current treatment options. Such patients are encountered in highly prevalent conditions such as viral infection and chronic rhino-sinusitis but also idiopathic, post-traumatic or toxic olfactory impairment [34]. Therefore, establishing a safe and efficient olfactory implantation procedure for long-term neuromodulation of the olfactory pathway is of paramount importance before any pilot study in affected patients.

Previous cadaveric studies evaluated transnasal transcribriform approaches as it seemed a more direct way to reach the OB [2, 26]. However, these endoscopic endonasal approaches carry a higher risk of CSF leak (25.5% versus 6.3% in transcranial approaches) and therefore, in the context of implantation, a potentially higher risk of subsequent infection and extrusion [29]. Intraoperative arterial injuries are also more frequent in endonasal endoscopic approaches (4.89% versus 1.86% in transcranial approaches) [28]. However, these data have been reported for endoscopic endonasal approaches in meningioma surgery and may not directly apply to the less invasive procedures envisioned for olfactory implantation. Given the absence of human cases, such risks remain hypothetical and should be balanced against those inherent to transcranial approaches. Regarding deep brain stimulation, for instance, the main complications comprise infections (2.8%), intracerebral hemorrhage (2.3%), and hardware-related problems such as wire fracture or disconnection (0.7%) [16], whereas surgical site infections following craniotomy were observed in 1.1% of patients [6]. In case of device failure, first implantation and explantation should not damage the OB and the OT in order to allow secondary implantations: such explantation and reimplantation would be more difficult through endoscopic endonasal approaches as secondary skull base reconstruction would be required. Moreover, given that the optimal site of the olfactory pathways for neurostimulation remains undefined in the literature [34], we also sought to explore less invasive or lower-risk approaches. For these reasons, our group has favored a minimally-invasive and routinely performed transcranial approach, the SKC, of which the most common morbidity is inadvertent frontal sinus intraoperative opening in case of highly pneumatized frontal sinus [32].

Our study has explored a previously unreported target for olfactory implantation, namely the OT. Anatomically speaking, the OT is less variable than the OB in terms of shape and dimensions [24, 30, 35], which would likely improve reproducibility of electrode placement between its ventral side and the planum sphenoidale. Moreover, access to the OB highly depends on the orbit roof bulging and the olfactory groove depth, which can reach up to 14 mm according to the Keros classification [1]. Accordingly, our study demonstrated an easier, quicker, more stable and likely safer implantation to the ventral OT compared to the dorsal OB. Another option would have been to insert the electrode into the dorsal OT (or even around the OT with a cuff electrode), but dissecting the upper aspect of the OT along the olfactory sulcus from the frontobasal lobe would not only require additional dissection time but also involve manipulation of the OT itself, which could compromise it directly or even more indirectly by putting at risk its microvascularization, potentially leading to its devascularization [11]. Functionally speaking, electrical stimulation of either the OB or the OT would lead to smell sensation in humans according to Kumar et al. [18], as both activate secondary olfactory neurons (mitral and tufted cells). These neurons have their cell bodies in the OB and send their axons, through the OT, to the PC and the other secondary olfactory areas [24, 25]. Therefore, stimulating either the OB or the OT should produce a similar outcome, including activation of the limbic system.

Another challenge of olfactory stimulation is to elicit patterns that able distinct olfactory perception. Although the exact mechanisms of olfactory coding are still being investigated, studies in rodents have demonstrated a high level of spatiotemporal organization within the OB. Different odorants elicit distinct maps of activity in the glomerular layer that likely translate into spatial activity at the level of mitral/tufted cells layers [10, 33]. Therefore, at least in rodents, secondary neurons are zonally organized at the level of the OB. An important question that remains unanswered is whether this organization is maintained along the OT. In our opinion, given that the anterior OT (first third) is as large as the OB and flat, it may be a better stimulation target than the posterior OT which is narrower and rounder: distinct patterns would be easier to obtain if axon bundles are spread rather than grouped [24, 30].

**Fig. 5:**
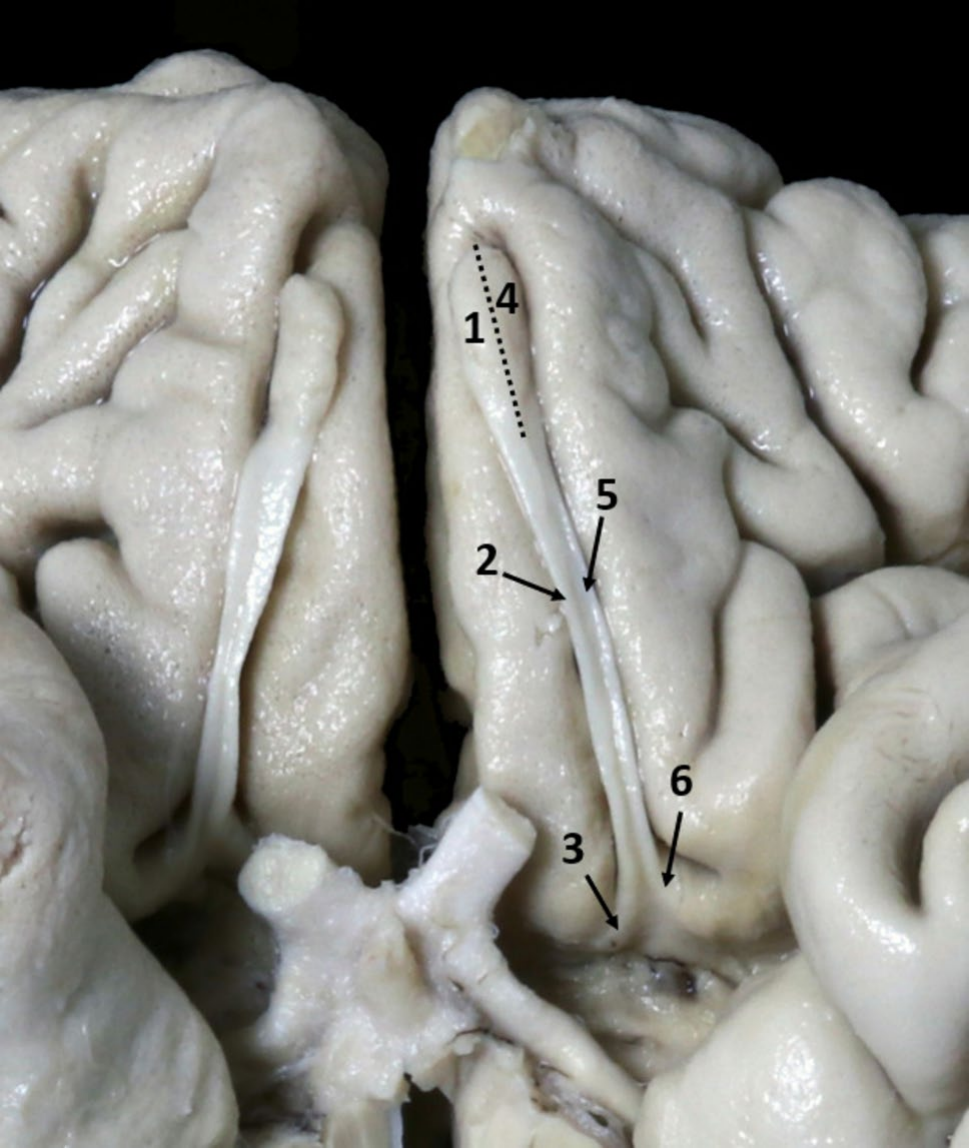
Illustration of the anatomical divide within the olfactory bulb [19] and the ventral olfactory tract, up to the olfactory striae. *1: Medial olfactory bulb, 2: Medial olfactory tract, 3: Medial olfactory stria, 4: Lateral olfactory bulb, 5: Lateral olfactory tract, 6: Lateral olfactory stria*

Moreover, a sulcus divides the OT into two tracts on its ventral side: the medial olfactory tract (MOT) and the lateral olfactory tract (LOT) as shown in Fig. 5. This is supported by a recent MRI tractography study that demonstrated a division in the OB, the medial OB (MOB) receiving ORNs from the upper nasal septum and the lateral OB (LOB) receiving ORNs from the middle and superior turbinate [19]. Finally, the posterior part of the OT separates to form the olfactory trigone which consists of 3 olfactory striae: the medial olfactory stria coming from the MOT, the lateral olfactory stria coming from the LOT and the thinner intermediate olfactory stria (coming from branches of both MOT and LOT) [9]. This anatomical detail could have significant importance in terms of OT neurostimulation as each of these striae has different targets and therefore, it can be hypothesized that stimulation of the LOT and MOT might have different outcomes. Indeed, according to anatomical and diffusion MRI studies in humans: (i) the lateral olfactory stria is connected to the PC and the amygdala (and probably the entorhinal cortex), (ii) the medial olfactory stria is connected to the anterior olfactory nucleus (and possibly the septal area of the limbic system) and might cross to the opposite side through the anterior commissure and (iii) the intermediate olfactory stria is connected to the olfactory tubercle [8, 9]. Therefore, further experiments are required to understand the functional impact of stimulating different areas of the OB and OT with a multi-electrode array, in sync with breathing to mimic natural olfaction.

This study has several limitations that might moderate our conclusions: the limited number of cadavers dissected, the use of a single type of electrode array preventing comparison with different electrode designs and the lack of CSF simulation that would provide a more realistic evaluation of electrode stability. In our opinion, deployment of a larger electrode over the planum sphenoidale, in contact with the ventral side of one or even both OTs, may increase electrode stability. However, it is important to note that the evaluation of electrode mobility is still challenging outside real-life implantation conditions, where physiological and mechanical constraints are fully at play.

## Conclusion and future perspectives

This study assessed for the first time a transcranial olfactory implantation technique that would be implemented in the context of olfactory loss related to damage to the OM or the olfactory filaments. It demonstrated that endoscopic ventral OT or dorsal OB implantation can be achieved through a SKA. However, OT implantation was easier and quicker, as orbital roof drilling was not necessary, and most importantly provided better electrode stability in comparison to dorsal OB implantation. Further clinical studies are needed to determine whether electrical stimulation of the OT using a multi-electrode array is indeed effective in eliciting distinct smell percepts, depending on the combination of activated contacts. These pilot studies evaluating short-term OT implantation could benefit from the use of commercially available implants such as the ABI.

## Data Availability

Iconography (photos, videos) of dissections is available.
